# Comparison of clinical outcomes between metallic and polymeric ureteral stents in malignant ureteral obstruction: a retrospective comparative study

**DOI:** 10.1186/s12894-026-02062-z

**Published:** 2026-01-30

**Authors:** Tomohiro Nishi, Ryuto Nakazawa, Yuki Morimoto, Ryuji Yamada, Hikaru Tsukada, Daisuke Shirai, Naoto Yoza, Koichiro Aida, Eiji Kikuchi

**Affiliations:** Department of Urology, St Marianna University School of Medicine, 2-16-1 Sugao, Miyamae-ku, Kawasaki, Kanagawa 216-8511 Japan

**Keywords:** Malignant ureteral obstruction, Metallic ureteral stent, Resonance, Polymeric ureteral stent, Peritoneal dissemination

## Abstract

**Background:**

The present study compared the clinical outcomes and indications of metallic ureteral stents (MS) and polymeric ureteral stents (PS) in patients with malignant ureteral obstruction (MUO).

**Methods:**

We analyzed 148 patients (240 ureters) with MUO who underwent ureteral stent placement at our Department of Urology between December 2014 and April 2022. The cohort included 67 patients (112 ureters) who received metallic stents (MS group) and 81 patients (128 ureters) who received polymeric stents (PS group). We evaluated overall survival and the primary underlying malignancies, and compared operative times, ureteral stent patency rates, and factors associated with stent obstruction between the two groups.

**Results:**

The one-year overall survival rate of patients with MUO was 27.2%, with a median survival time of 209 days. The main primary malignancies were gynecologic and gastrointestinal cancers, most commonly cervical, gastric, colorectal, breast, and ovarian cancers, in that order. The operative time for stent insertion was significantly longer in the MS group than in the PS group for both bilateral (*p* = 0.0004) and unilateral (*p* = 0.0094) placements. The one-year stent patency rate was significantly higher in the MS group (62.0%) than in the PS group (48.5%) (*p* = 0.0144). Factors associated with stent obstruction included lower ureteral obstruction (*p* = 0.0401), direct tumor compression (*p* = 0.0172), pyuria (*p* = 0.0028), and elevated preoperative serum creatinine (*p* = 0.0088) in the MS group, and peritoneal dissemination (*p* = 0.0005) in the PS group. A comparison of stent patency between the groups according to obstruction factors showed no significant differences for lower ureteral obstruction (*p* = 0.5140), direct tumor compression (*p* = 0.8215), or pyuria (*p* = 0.8401). However, among patients with peritoneal dissemination, the stent patency period was significantly longer in the MS group (*p* = 0.0001).

**Conclusions:**

Metallic ureteral stenting, which has higher patency rates than PS, is a safe and effective treatment option for MUO, particularly in the patients with peritoneal dissemination.

## Introduction

In urological practice, ureteral stent placement is often performed to relieve postrenal acute kidney injury (AKI) caused by malignant ureteral obstruction (MUO), in which ureters are extrinsically compressed by tumors originating from organs outside the urogenital tract. However, when severe ureteral stenosis results from tumor compression or when the ureteral orifice cannot be identified due to bladder invasion, ureteral stent placement becomes technically difficult, and percutaneous nephrostomy is selected instead [1].

The main reasons ureteral stent placement is generally preferred as the first-line intervention are its ease of management and the ability to maintain body image. Ureteral stents are managed by transurethral replacement every 3–6 months, whereas nephrostomy catheters require monthly exchanges, making their management more complex. Furthermore, since the catheter remains exposed outside the body, nephrostomy significantly impairs body image. Although nephrostomy provides reliable urinary drainage and is superior in ensuring the decompression of urinary tract obstruction, it generally reduces patients’ quality of life (QOL). Therefore, many urologists prefer ureteral stenting as the initial treatment option whenever feasible [2].

On the other hand, conventional polymeric ureteral stents (PS) have the unresolved issue of stent failure caused by tumor compression. Consequently, there has been a growing need for new treatment options that ensure long-term urinary drainage while avoiding percutaneous nephrostomy. As one solution, the metallic ureteral stent (MS) (Cook Resonance^®^; Cook Medical, Bloomington, IN, USA) was covered by the Japanese national health insurance system from December 2014, making it available for clinical use. MS is composed of a cobalt–nickel–chromium–molybdenum alloy and exhibits greater resistance to external compression than PS [3]. Therefore, MS is expected to reduce the risk of stent failure and allow the avoidance of nephrostomy, and its safety and efficacy have already been reported [4]. In addition, since the maximum indwelling period of MS is 12 months, its exchange frequency is lower than that of PS, resulting in better cost-effectiveness and a reduced financial burden for patients [5, 6].

Patients with MUO often face not only the challenge of preventing stent failure, but also the difficult decision of whether to continue anticancer therapy or transition to palliative care. Since the median survival time after the diagnosis of MUO is generally less than one year [7–9], maintaining QOL while meeting patients’ preferences and goals is crucial in both cancer management and end-of-life care.

Therefore, the present study retrospectively compared clinical outcomes and stent patency rates between MS and PS cases at our department to clarify the appropriate selection of ureteral stents for MUO and identify the optimal indications according to patient backgrounds and disease characteristics.

## Methods

### Patient characteristics

This study included 148 patients (240 ureters) with MUO who underwent ureteral stent placement at our department between December 2014 and April 2022. The cohort comprised 67 patients (112 ureters) who received MS (MS group) and 81 patients (128 ureters) who received PS (PS group). Among those who received MS, 45 patients (80 ureters) had previously undergone PS pre-stenting. We analyzed the overall survival and primary underlying malignancies of patients with MUO and compared operative times, stent patency rates, factors associated with obstruction, and stent patency according to obstruction factors between the two groups. Patients diagnosed with MUO in whom ureteral stent placement was unsuccessful and who subsequently required percutaneous nephrostomy, those lost to follow-up during the observation period, and patients who had different types of ureteral stents placed bilaterally were excluded to maintain consistency in the analysis.

### Surgical technique

Stent placement and exchange were performed under general or spinal anesthesia, with the patient in the lithotomy position, using fluoroscopic guidance and transurethral cystoscopy. All stents had a diameter of 6 Fr and a length of 20–26 cm. All ureteral stents were inserted retrogradely; no cases required antegrade placement. Retrograde pyelography was performed under fluoroscopy, and PS was then placed retrogradely using a standard technique with a guidewire, while MS was placed using a retrograde technique with an outer sheath, inner sheath, and guidewire. After removing the guidewire and inner sheath, the inner sheath was reinserted as a pusher into the outer sheath, and the proximal end of the stent was confirmed fluoroscopically to be positioned in the renal pelvis. The outer sheath was then withdrawn, and the distal end of the stent was confirmed to be positioned in the bladder under both cystoscopic and fluoroscopic observations.

### Follow-up

Postoperative evaluations were performed using serum creatinine (SCr) measurements, plain abdominal radiography, computed tomography (CT), and ultrasonography. The timing of these assessments was selected by the attending physician at the outpatient clinic. Stent failure was defined as the development of postrenal AKI regardless of the presence or absence of hydronephrosis, or as any event requiring stent replacement or conversion to nephrostomy due to urinary tract infection (UTI) or other severe complications.

### Statistical analysis

Overall survival rates, stent patency rates, and stent patency periods according to obstruction factors were analyzed using the Kaplan–Meier method and evaluated with the Log-rank test. Sex and predictors of stent obstruction were compared between groups using Pearson’s chi-square test, while age and operative times were compared using the Wilcoxon test. A p-value < 0.05 was considered to be significant. All statistical analyses were performed using JMP Student Edition version 18.2.1 (SAS Institute Inc.). No a priori power or sample size calculation was performed due to the retrospective nature of the study.

## Results

### Patient characteristics

The baseline characteristics of patients in the MS and PS groups are summarized in Table 1. The underlying causes of MUO were diverse, with gynecologic and gastrointestinal malignancies being the most common. The most frequent primary cancers were cervical, gastric, colorectal, breast, and ovarian cancers, in that order. The mean operative time was significantly longer in the MS group than in the PS group for both bilateral (*p* = 0.0004) and unilateral (*p* = 0.0094) stent placements. Regarding complications, UTI were more frequent in the MS group, and in three of these cases, the stents were subsequently replaced with PS. One case of stent dislodgement occurred in the MS group. No cases of gross hematuria or retroperitoneal urinary leakage were observed in either group. Bladder irritation symptoms were infrequent and comparable between the two groups.

**Table 1:** Patient characteristics

Characteristics	Metallic stent group	Polymeric stent group	*p*-value
Patients, n	67	81	
Male/Female, n	17/50	24/57	0.5647
Age (years), mean ± SD	65.6 ± 11.9	62.1 ± 13.2	0.0835
Bilateral/Unilateral, n	45/22	47/34	
Operative time (min), mean ± SD			
Bilateral	45.3 ± 15.4	34.8 ± 16.8	**0.0004**
Unilateral	30.9 ± 12.5	22.5 ± 10.0	**0.0094**
Complications, n (%)			
Urinary tract infection	12 (17.9)	5 (6.2)	
Bladder irritability	5 (7.5)	2 (2.5)	
Migration	1 (1.5)	0 (0.0)	
Causes of MUO, n (%)			
Uterine cancer	18 (26.9)	21 (25.9)	
Gastric cancer	13 (19.4)	17 (21.0)	
Colorectal cancer	10 (14.9)	11 (13.6)	
Breast cancer	10 (14.9)	8 (9.9)	
Ovarian cancer	7 (10.4)	6 (7.4)	
Malignant lymphoma	4 (6.0)	6 (7.4)	
Occult cancer	1 (1.5)	3 (3.7)	
Others	4 (6.0)	9 (11.1)	
Ureters, n	112	128	
Left/Right, n	54/58	59/69	

*P*-values less than 0.05 are shown in bold

### Overall survival of MUO

**Fig. 1:**
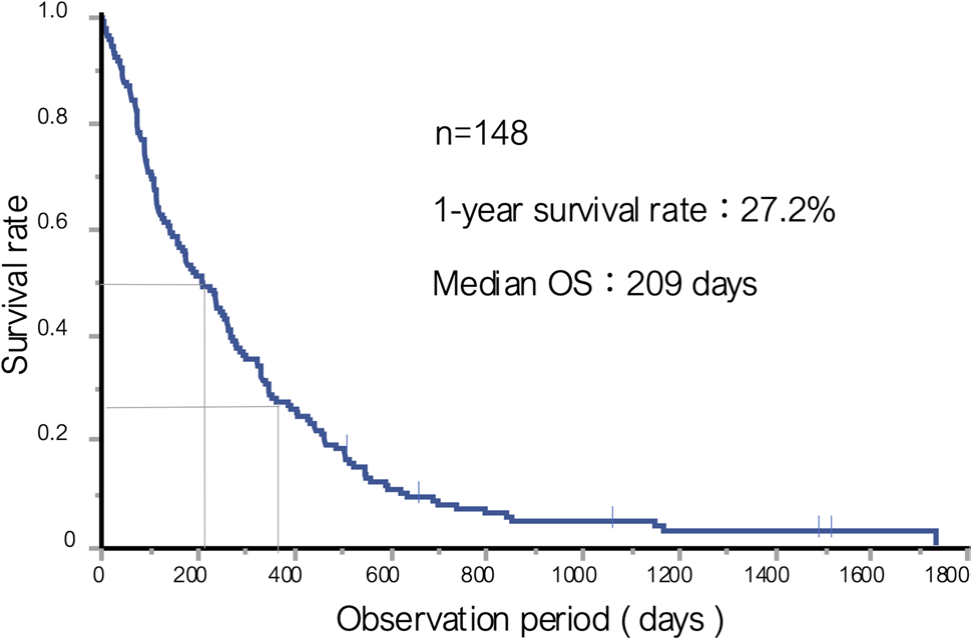
Overall survival of patients with malignant ureteral obstruction. A Kaplan–Meier curve of overall survival for all patients with MUO during the observation period

The one-year overall survival rate for patients with MUO was 27.2%, and the median survival time was 209 days (Fig. 1).

### Patency rate of ureteral stents in each group

**Fig. 2:**
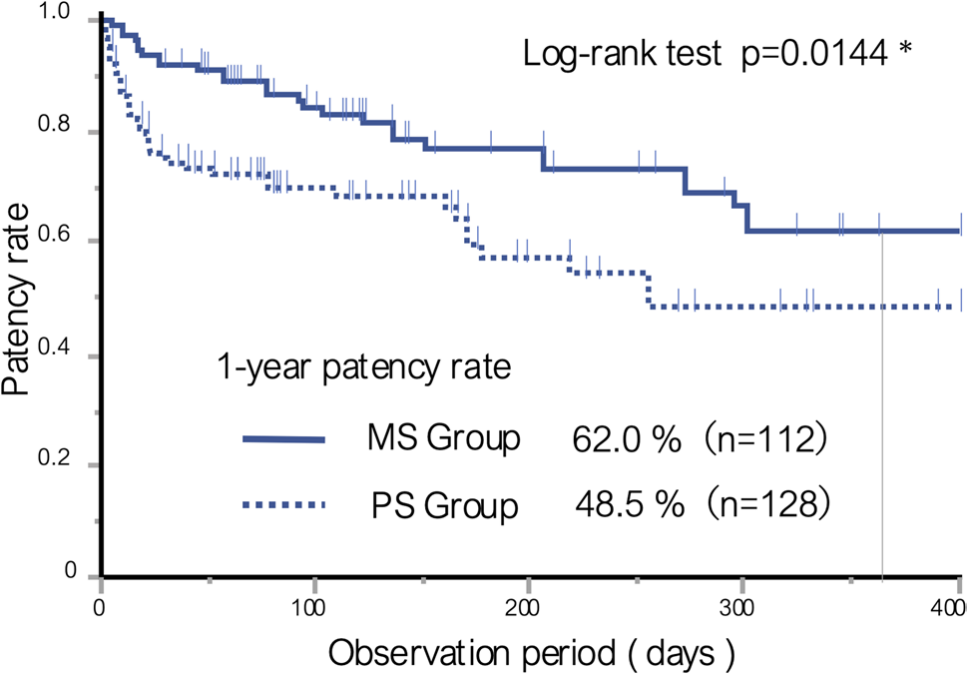
Ureteral stent patency rates in metallic stent and polymeric stent groups. Kaplan–Meier curves of ureteral stent patency during the observation period for the MS group (solid line) and PS group (dotted line)

The one-year patency rates of ureteral stents were 62.0% in the MS group and 48.5% in the PS group (Fig. 2), being significantly higher in the former (*p* = 0.0144).

### Analysis of factors associated with ureteral stent obstruction

**Fig. 3:**
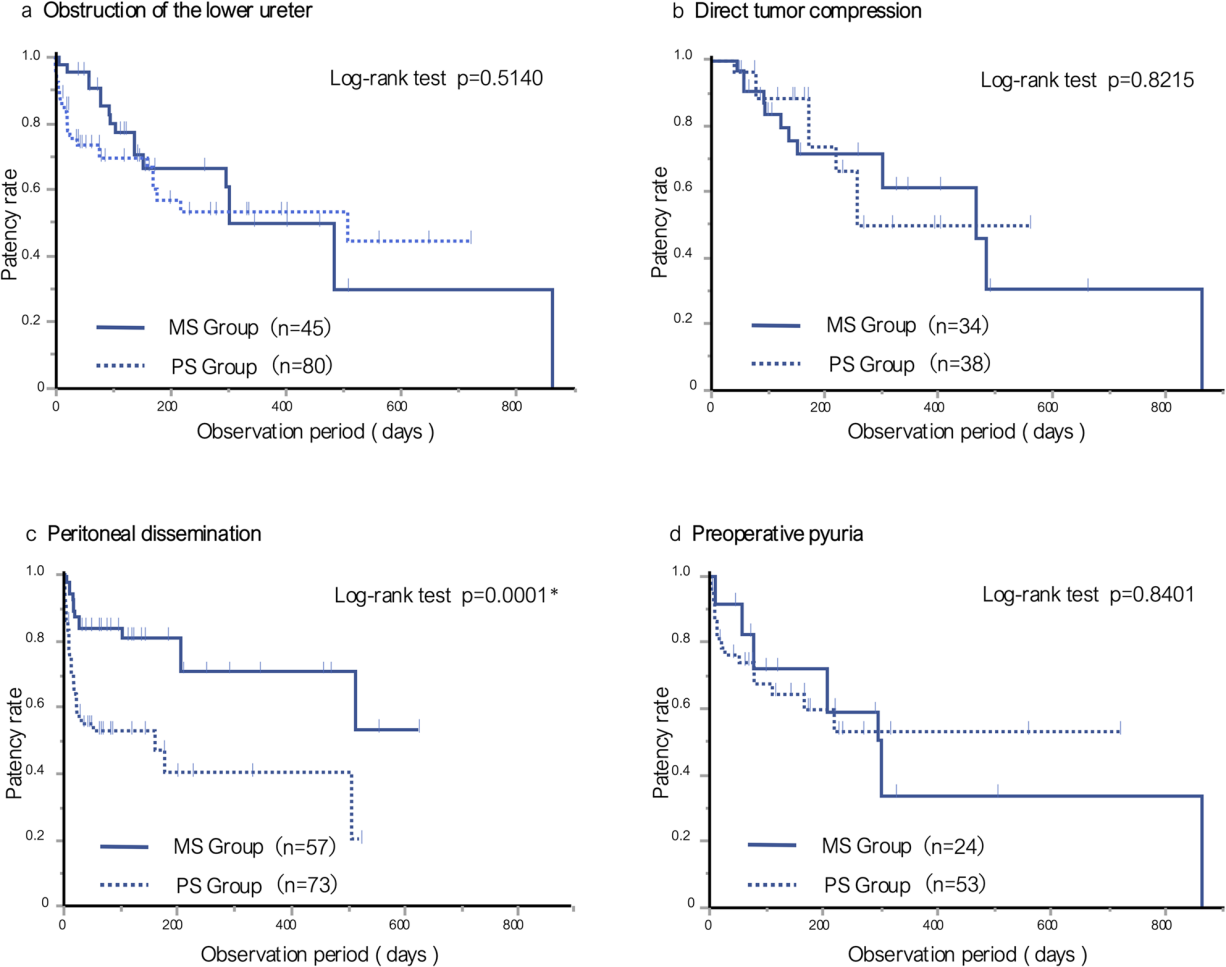
Kaplan–Meier curves of ureteral stent patency according to obstruction-related factors in both groups. a-d. Kaplan–Meier curves of ureteral stent patency during the observation period for the MS group (solid line) and PS group (dotted line) according to obstruction-related factors: **a** lower ureteral obstruction, **b** direct tumor compression, (**c**) peritoneal dissemination, and (**d**) preoperative pyuria. A significant difference was observed for (**c**), but not for (**a**), (**b**), or (**d**)

The results of the analysis of factors associated with ureteral stent obstruction are summarized in Table 2. In the MS group (Table 2a), peritoneal dissemination correlated with stent patency (*p* = 0.0497). Conversely, lower ureteral obstruction (*p* = 0.0401), direct tumor compression (*p* = 0.0172), preoperative pyuria (*p* = 0.0028), and elevated preoperative SCr (*p* = 0.0088) correlated with stent obstruction. In the PS group (Table 2b), direct tumor compression (*p* = 0.0299) and lymph node metastasis (*p* = 0.0301) correlated with stent patency, whereas peritoneal dissemination correlated with stent obstruction (*p* = 0.0005). When stent patency periods were compared between groups according to these obstruction-related factors using the Kaplan–Meier method, a significantly higher patency rate was observed in the MS group only in patients with peritoneal dissemination (*p* = 0.0001; Fig. 3c). No significant differences were found between groups for other factors (Fig. 3a, b, d).

**Table 2:** Analysis of factors associated with stent patency and obstruction in ureteral units of patients with malignant ureteral obstruction

a) Metallic Stent Group			*n* (%)
	Patency	Obstruction	*p*-value
Bilateral	61 (79.2)	29 (82.9)	0.6534
Area of obstruction			
Upper	27 (35.1)	7 (20.0)	0.1080
Middle	21 (27.3)	8 (22.9)	0.6210
Lower	26 (33.8)	19 (**54.3**)	**0.0401**
Pathology of obstruction			
Direct tumor compression	18 (23.4)	16 (**45.7**)	**0.0172**
Lymph node	15 (19.5)	6 (17.1)	0.7689
Peritoneal dissemination	44 (**57.1**)	13 (37.1)	**0.0497**
Preoperative pyuria	11 (16.2)	13 (**44.8**)	**0.0028**
Radiotherapy (+)	20 (26.0)	15 (42.9)	0.0740
Median basal Cr > 0.72 mg/dl	35 (46.1)	22 (62.9)	0.0998
Median preoperative Cr > 1.4 mg/dl	34 (44.7)	25 (**71.4**)	**0.0088**

b) Polymeric stent group			*n (%)*
	Patency	Obstruction	*p*-value
Bilateral	58 (69.9)	34 (75.6)	0.4953
Area of obstruction			
Upper	12 (14.5)	3 (6.7)	0.1907
Middle	19 (22.9)	14 (31.1)	0.3101
Lower	52 (62.7)	28 (62.2)	0.9616
Pathology of obstruction			
Direct tumor compression	30 (**36.1**)	8 (17.8)	**0.0299**
Lymph node	15 (**18.1**)	2 (4.4)	**0.0301**
Peritoneal dissemination	38 (45.8)	35 (**77.9**)	**0.0005**
Preoperative pyuria	35 (55.6)	18 (47.4)	0.4248
Radiotherapy (+)	24 (28.9)	20 (44.4)	0.0774
Median basal Cr > 0.72 mg/dl	39 (48.2)	19 (45.2)	0.7592
Median preoperative Cr > 2.1 mg/dl	45 (54.9)	24 (53.3)	0.8672

*P*-values less than 0.05 and variables with significantly higher proportions between the patent and obstructed groups are shown in bold

## Discussion

Ureteral stent placement is a common clinical procedure performed to preserve renal function in cases of postrenal AKI caused by ureteral obstruction due to various benign or malignant diseases. In patients with MUO, progressive compression or obstruction of the ureter often occurs as the underlying malignancy advances, and previous studies indicated that 35–45% of patients experience stent failure after PS placement [10]. Consequently, PS frequently leads to obstruction, often necessitating conversion to percutaneous nephrostomy.

Extensive efforts have been made to improve the patency of ureteral stents in MUO patients through modifications in stent design and materials. The MS (Cook Resonance^®^) used in the present study has a highly rigid structure without an inner lumen; it consists of a tightly coiled metal design that allows urine drainage through intercoil gaps. This unique structure differs from that of PS and provides high resistance to external compression, which is considered to contribute to superior stent patency.

Complications associated with MS placement include gross hematuria, bladder irritation, UTI, dysuria, pain, encrustation, and subcapsular renal hematoma. Previous studies reported that the frequency of these complications did not markedly differ from that of PS [11, 12]. In our institution, as shown in Table 1, no MS-specific complications were observed. Since the insertion technique for MS differs from that for PS, the operative time is generally longer [13]. In the present study, the operative time for MS placement was significantly longer than that for PS. However, no increase in adverse events related to a longer operative time was observed, suggesting that MS may be used as safely as PS.

In the present study, the one-year stent patency rate was significantly higher in the MS group (62.0%) than in the PS group (48.5%). Goldsmith et al. reported a one-year patency rate of 65% for MS in patients with MUO [8], which is largely consistent with the present results. Furthermore, Chow et al. reported that MS provides approximately four months longer patency than PS [14]. Collectively, these findings suggest that MS offers superior stent patency to PS in the management of MUO.

In the analysis of factors associated with stent obstruction, lower ureteral obstruction, direct tumor compression, preoperative pyuria, and elevated preoperative SCr were identified as significant risk factors in the MS group. Previous studies reported several predictors of MS obstruction, including UTI, elevated preoperative SCr, the presence of lower gastrointestinal cancer, ureteral obstruction at the abdominal level, prior radiation therapy, lymph node metastasis, and bilateral ureteral obstruction [7, 15, 16]. The present results are consistent with these findings. Regarding the relationship between preoperative SCr and MS patency, some studies found that replacing PS with MS after the normalization of SCr resulted in better patency outcomes [17], and others reported that when preoperative SCr was below 2.0 mg/dL, a longer stent patency period may be expected [14]. Taken together, these findings and the present results indicate that elevated preoperative SCr is associated with a higher risk of MS obstruction. Furthermore, Brown et al. found that patients with UTI had a significantly higher rate of MS obstruction and that the presence of preoperative pyuria increased the risk of obstruction [18]. This may be explained by the structural characteristics of MS, which lacks an inner lumen and, thus, provides slower urinary drainage [19, 20]. Accordingly, MS placement needs to be avoided in patients with postrenal AKI who have elevated preoperative SCr or preoperative pyuria. Peritoneal dissemination was associated with stent patency in the MS group and with obstruction in the PS group. Notably, comparisons of the duration of stent patency between the two groups revealed that the patency period was significantly longer in patients with peritoneal dissemination in the MS group. These results suggest that MS is more suitable than PS for managing ureteral obstruction caused by peritoneal dissemination. Peritoneal dissemination often leads to continuous ureteral narrowing due to strong extrinsic compression, and previous studies reported that PS, which has lower mechanical strength, was prone to obstruction under these conditions [21]. The present results are consistent with these findings.

**Fig. 4:**
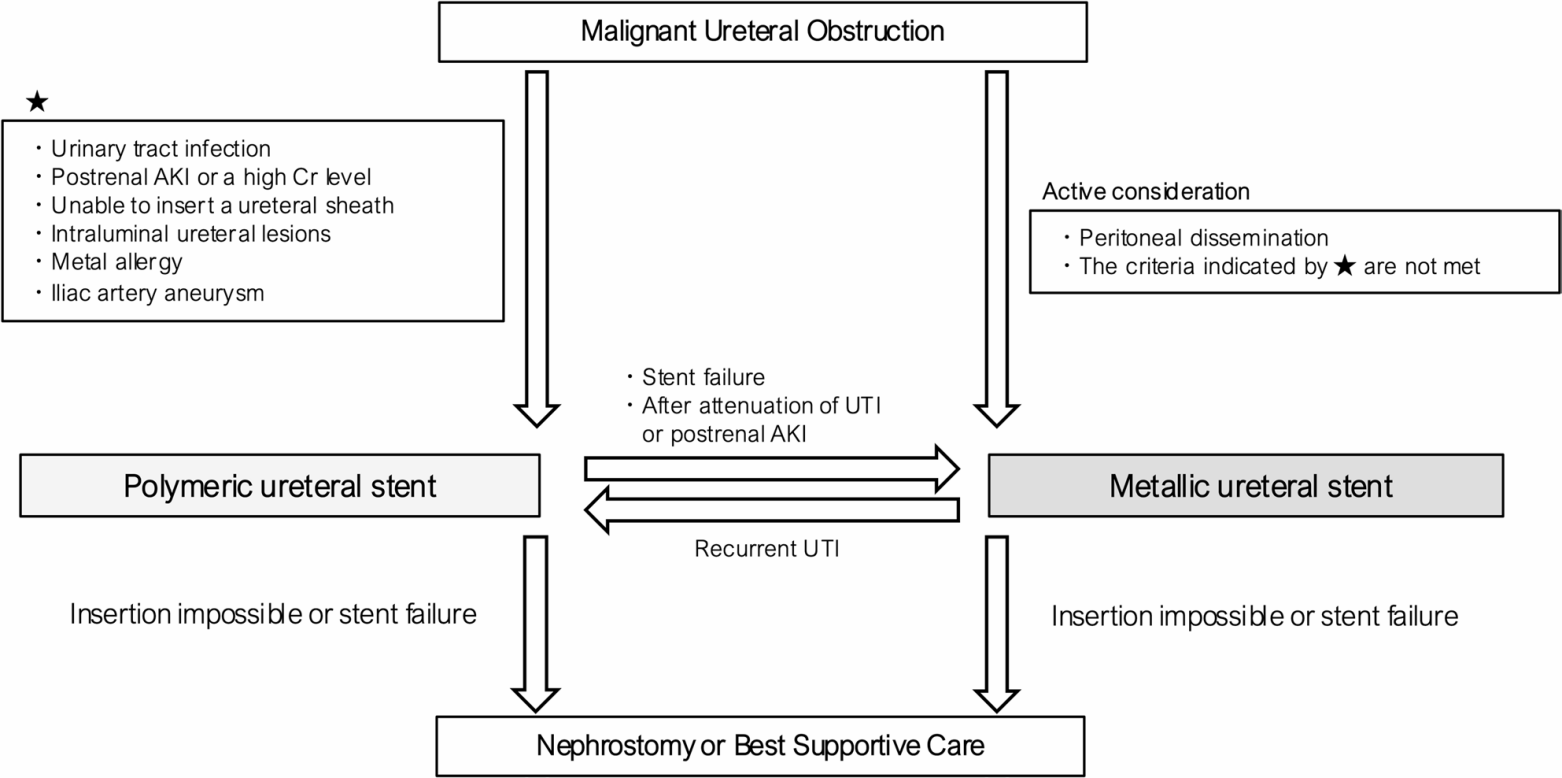
Flowchart for ureteral stent selection in patients with malignant ureteral obstruction

Based on these findings, we developed a flowchart for selecting ureteral stents in patients with MUO at our institution (Fig. 4). As an initial assessment, PS needs to be selected as the first-line option for patients presenting with UTI or postrenal AKI accompanied by elevated SCr, for whom prompt urinary drainage is required. In cases with the attenuation of UTI or normalization of SCr, the conversion to MS needs to be considered. PS also needs to be selected for patients with severe ureteral stenosis preventing the insertion of a ureteral access sheath, the presence of intraluminal ureteral lesions, or a history of metal allergy. In addition, when a common iliac artery aneurysm is present, PS needs to be selected because MS placement has been reported to cause ureteroarterial fistula formation [22]. Conversely, in patients without these contraindications and with ureteral obstruction due to peritoneal dissemination, MS needs to be considered as the first-line option.

To the best of our knowledge, previous comparisons of the clinical outcomes of MS and PS did not include as many cases as the present study, underscoring its clinical relevance. The selection of the appropriate type of ureteral stent based on individual disease characteristics and patient backgrounds is expected to contribute to longer stent patency and improved QOL. Since MUO often causes AKI and UTI that may compromise cancer treatment, and because its median survival time is less than one year, a rapid intervention and appropriate device selection are critically important in managing this highly urgent condition.

### Limitations

This study has several limitations. First, this was a retrospective, single-center study with a limited sample size, which may have resulted in insufficient statistical power. Therefore, the findings of this study should be interpreted as hypothesis-generating, and future validation in larger prospective studies is warranted. Second, the selection of polymeric or metallic ureteral stents was based on the attending physician’s discretion and patient preference, and treatment allocation was not randomized, which may have introduced selection bias. Third, due to the retrospective design, confounding factors may have influenced treatment selection and clinical outcomes. Finally, patients who required percutaneous nephrostomy due to unsuccessful stent placement were excluded; thus, our results may not be directly applicable to patients with more severe malignant ureteral obstruction.

## Conclusions

This study demonstrated that MS was safely used in patients with MUO and had a higher patency rate than PS, particularly in the patients with peritoneal dissemination. A comprehensive understanding of patient backgrounds, disease characteristics, and the specific features of each type of ureteral stent is essential. Continuous efforts are needed to improve QOL in patients with MUO through appropriate device selection.

## Data Availability

The datasets used and/or analyzed during the current study are available from the corresponding author on reasonable request.

## Abbreviations

MS: Metallic ureteral stent
PS: Polymeric ureteral stent
MUO: Malignant ureteral obstruction
AKI: Acute kidney injury
QOL: Quality of life
OS: Overall survival
UTI: Urinary tract infection
SCr: Serum creatinine
